# Rett syndrome in Ireland: a demographic study

**DOI:** 10.1186/s13023-024-03046-8

**Published:** 2024-01-31

**Authors:** Komal Zade, Ciara Campbell, Snow Bach, Hazel Fernandes, Daniela Tropea

**Affiliations:** 1 Department of Psychiatry, School of Medicine, Trinity Centre for Health Sciences, Trinity College Dublin, St James Hospital, Dublin, D08 W9RT Ireland; 2 Neuropsychiatric Genetics, Department of Psychiatry, School of Medicine, Trinity College Dublin, Trinity Translational Medicine Institute, St James’s Hospital, Dublin, Ireland; 3 Trinity College Institute of Neuroscience, Trinity College Dublin, Dublin, Ireland; 4 FutureNeuro, The SFI Research Centre for Chronic and Rare Neurological Diseases, Dublin, Ireland; 5 Consultant Child and Adolescent Psychiatrist, Barnet, Enfield and Haringey Mental Health NHS Trust, London, UK

**Keywords:** Rett syndrome, *MECP2* gene, Neurodevelopmental disorder

## Abstract

**Background:**

Rett syndrome (RTT) is a rare neurodevelopmental condition associated with mutations in the gene coding for the methyl-CpG-binding protein 2 (*MECP2*). It is primarily observed in girls and affects individuals globally. The understanding of the neurobiology of RTT and patient management has been improved by studies that describe the demographic and clinical presentation of individuals with RTT. However, in Ireland, there is a scarcity of data regarding individuals with RTT, which impedes the ability to fully characterize the Irish RTT population. Together with the Rett Syndrome Association of Ireland (RSAI), we prepared a questionnaire to determine the characteristics of RTT individuals in Ireland. Twenty-five families have participated in the study to date, providing information about demographics, genetics, familial history, clinical features, and regression.

**Results:**

The results show that Irish individuals with RTT have comparable presentation with respect to individuals in other countries; however, they had a better response to anti-epileptic drugs, and fewer skeletal deformities were reported. Nonetheless, seizures, involuntary movements and regression were more frequently observed in Irish individuals. One of the main findings of this study is the limited genetic information available to individuals to support the clinical diagnosis of RTT.

**Conclusions:**

Despite the limited sample size, this study is the first to characterize the RTT population in Ireland and highlights the importance of having a swift access to genetic testing to sharpen the characterization of the phenotype and increase the visibility of Irish individuals in the international RTT community.

**Graphical abstract:**

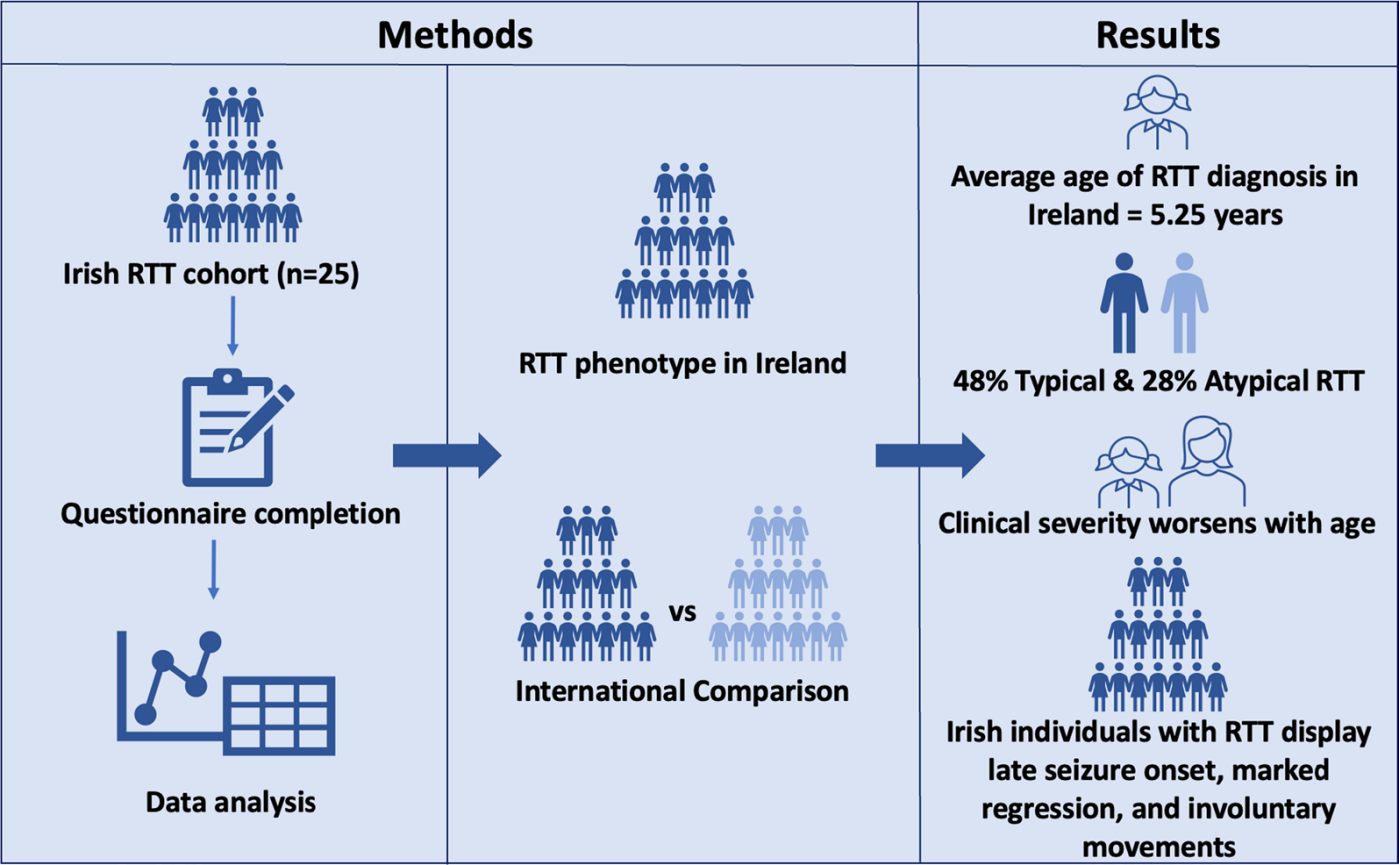

## Background

Rett syndrome, originally described in the 1960’s by Andreas Rett, is an X-linked rare neurodevelopmental condition with specific clinical features [1]. It affects brain function and development, with global prevalence ranging from 5–10 cases (95% CI) per 100,000 females [2]. After an apparently typical developmental period of approximately 6 months, individuals with classic RTT present with regression of acquired developmental skills at 1–4 years of age [3]. There are four stages of RTT defining the clinical course: (i) early onset: birth to 6–18 months, (ii) rapid developmental deterioration: 1–4 years, (iii) plateau/pseudo-stationary: 4–10 years, and (iv) late motor deterioration: 10–30 years [3].

Symptoms of RTT can range in clinical severity and include acquired hypotonia and abnormal deceleration in rate of head growth observed at approximately 3–6 months of age. Loss of previously acquired linguistic skills, poor muscle tone, slower cerebral and physical development, jerky limb movements, and behavioral and emotional dysregulation, such as restricted sociability, are observed at approximately 1–4 years of age. Stereotypic hand movements are a defining feature of RTT. Other symptoms include seizures, bruxism, apraxia, cold hands and feet, gastrointestinal (GI) issues such as bloating, cardiac and breathing issues (mainly episodic hyperventilation, breath-holding, and Valsalva manoeuvres), forced saliva and air expulsion, sleep disturbances, and musculoskeletal abnormalities (predominantly scoliosis and lower limb deformities) [4, 5]. Autistic traits are present, but only in stage 2 [6].

Most individuals with RTT are females and have mutations in the gene coding for Methyl-CpG binding protein 2 (*MECP2*) located on the Xq28 chromosome. Males with RTT survive for variable periods including those who present with classic RTT due to somatic mosaicism or Klinefelter syndrome [7, 8]. Hagberg and associates led pioneering work to develop clinical diagnostic criteria [1, 9–11]. Neul et al., revised the criteria in their paper in 2010 [12]. Their recommendations aimed to minimize clinical and genetic heterogeneity with regard to diagnosis and classification of RTT during clinical trials. The authors suggested that individuals with clinically definite typical RTT without a *MECP2* mutation should not be excluded from participation. Further, they suggested that neither should individuals with *MECP2* mutations and a clinical condition distinct from RTT be excluded from clinical trials. The authors recommended a consideration of a diagnosis of RTT when there is a noted deceleration of head growth. The main criteria apply to both typical and atypical RTT and is defined as partial or complete loss of acquired purposeful hand skills; partial or complete loss of acquired spoken language; gait abnormalities including dyspraxia or no ability to walk and stereotypic hand movements such as hand wringing/squeezing, clapping/tapping, mouthing, and washing/rubbing automatisms [12].

For a diagnosis of typical RTT, all the main and exclusion criteria must be met along with an observed period of regression, followed by recovery or stabilization. The authors defined exclusion criteria for typical RTT which is the absence of brain injury secondary to trauma (peri- or postnatally), neurometabolic disease, or severe infection that causes neurological problems and grossly abnormal psychomotor development in first 6 months of life. Supportive criteria are not required [12].

The diagnosis of atypical RTT is applicable when at least 2 out of the four main criteria and 5 out of the 11 supportive criteria are met and the individual has undergone a period of regression followed by recovery or stabilisation. Supportive criteria are identified as breathing disturbances when awake, bruxism when awake, impaired sleep pattern, abnormal muscle tone, peripheral vasomotor disturbances, scoliosis/kyphosis, growth retardation, small cold hands and feet, inappropriate laughing/screaming spells, diminished response to pain and intense eye communication—“eye pointing” [12]. Among atypical RTT cases, there is the preserved speech or "Zappella variant" (Z-RTT), which is defined by milder symptoms and a degree of speech ability. The ‘Rett-like’ subtype refers to conditions where many but not all the diagnostic criteria of RTT are fulfilled, and other causes and genetic mutations other than *MECP2* are associated with the clinical presentation [13]. The classification of RTT and its subtypes is continually evolving.

MeCP2 is a chromosome-binding protein that can act as transcriptional activator or repressor and controls miRNA function [14]. *MECP2* is important for brain development and function, but the protein is ubiquitously expressed in the body [15, 16]. Although over 90% of RTT individuals have mutations in the *MECP2* gene, there are individuals with a clinical diagnosis of RTT who do not have *MECP2* mutations. Suter and colleagues have also identified individuals with *MECP2* mutations who lack clinical features of Rett syndrome [17]. Recently, additional factors have been suggested to play a role in the development of the RTT phenotype [18]. In addition to *MECP2*, other genetic variants have been identified as contributing to RTT-like phenotypes, including *CDKL5* and *FOXG1, MEF2C, TCF4 *[19, 20]*, CTNNB1, WDR45* [21]*, ATP6V0A1, USP8, MAST3, and NCOR2* [22]*.*

Overall, there is significant clinical and phenotypic variability associated with RTT. The variation in RTT presentation is associated with several factors, including the nature of the *MECP2* mutation present [23–26], the degree of X-chromosome inactivation [27–29], the individual’s genetic background, and the site and location of MeCP2 expression in the individual’s brain [3]. Additional variability is reported in demographic analysis from specific populations in several countries, such as the USA and Canada [30–35]; Italy [4, 36]; Denmark [37–39]; Australia [7, 40–46]; UK [43, 47–49]; the Netherlands and Belgium [50, 51]; Brazil [52]; Poland [53]; Sweden [54]; and an international analysis [55]. Due to this degree of variability in presentation, RTT is commonly misdiagnosed and often difficult to treat effectively, especially when considering differences in the genetic basis of the condition. Barriers arising from the variability of RTT have led to the development of several RTT databases to categorize and analyse different RTT cohorts. These include- InterRett, the Australian Rett Syndrome Database (ARSD), the Rett Syndrome Natural History Study (USA), and the Rett Networked Database [56]. These databases have led to great advancements in understanding the biology of RTT and in recording the genotypic and phenotypic variation of RTT. They also contribute to recognizing the presence of different cohorts of patients.

## Methods

### Study design and data source

In collaboration with the RSAI, we prepared a questionnaire for caregivers outlining the demographic characteristics of RTT individuals in Ireland. The questions included were a subset of the questionnaire established by Grillo and colleagues, which were selected and adapted in partnership with the families affiliated with RSAI to create a user-friendly version that was comprehensible and easy to complete [56].

RSAI distributed questionnaires from May 2022, along with consent forms and a patient information leaflet [57]. Information on the purpose of the research and benefits of participation were also provided. In addition, the RSAI disseminated information through a prerecorded video and distributed the questionnaire to their additional members. After collecting the questionnaires, the RSAI removed personal information, assigned codes, and sent the anonymous data to the researchers for analysis, who were blinded to all personal information. The entire process complied with General Data Protection Regulation policies and was approved by the TCD Faculty of Health Science Ethical Committee (210404). Only participants who provided consent were included in the analysis. The questionnaire included 26 questions, which inquired about the following details: current age, height, and weight of the individual; age at diagnosis; place of diagnosis; clinician/service that performed the diagnosis; type of RTT; genetic test and mutation; regression (behavioural, speech, motor, growth failure, and regression of hand use); hand use of individual; presence of gastrointestinal (GI) problems; communication (verbal and/or nonverbal, and ability to understand/interact with others); motor skills of individual (ability to sit, walk, stand, walk with support); epilepsy status (seizures, age of seizure onset, current medications, resistance to anti-epileptic medications, family history of seizures, and details of the seizures); breathing abnormalities; bloating; cold extremities; sphincter control; skeletal abnormalities; involuntary movements; bruxism; age of pubertal onset (if applicable); additional diagnostic tests performed (EEG, cardiac activity, breathing activity, imaging); family history (consanguinity between parents, history of RTT/other diseases in the family, siblings and their age, sex); and mother’s pregnancy details (regular/irregular pregnancy, medications taken during the gestation period, regular/assisted delivery). For some questions, the participants were asked to choose the most commonly observed presentation from a list of options. For example, when asked about behavior, the options included excitement, sadness, self-injury, injury to others, anxiety, and difficulty falling asleep at night. A free-text space was left for participants to enter any additional comments that they felt were important to include in the questionnaire.

For comparisons with other populations, the data were acquired by a literature search using the search terms ‘Rett syndrome’, ‘RTT’, ‘phenotype’, ‘mutation’, ‘population’, and ‘international data’. Articles published from 1985 to 2023 were then chosen from a PubMed search. The nations in this comparison included the USA and Canada [30–35]; Italy [4, 36]; Denmark [37–39]; Australia [14, 40–46]; the UK [43, 47–49]; the Netherlands and Belgium [50, 51]; Brazil [52]; Poland [53]; Sweden [54]; and an international analysis [55]. The countries were represented using the ISO country codes: United States of America-US, Italy-IT, Denmark-DK, Netherlands-NL, Sweden-SE, Australia-AU, United Kingdom-UK, Brazil-BR, Poland-PL, and Ireland-IE; for the international cohort, we used the abbreviation ‘Intl’.

### Sample

The study sample included only the participants who answered the questionnaire during the study period and were recruited through RSAI. Analysis was conducted on all the questionnaires that were returned to the researchers at the time of data analysis and submission (n = 25). For the analysis, only answered responses were considered, as some questions were left unanswered. Each participant corresponds to one individual only. The comparison of RTT characteristics involves examining populations from various countries. The number of individuals with RTT in each respective country is juxtaposed with the overall population in Table 1.

**Table 1:** Comparison of number of Rett syndrome individuals studied across countries

Country	No. of individuals with RTT included in studies	Total population (million)	References
USA and Canada	919	331.9	[30–35]
Italy	84	59.11	[4, 36]
Denmark	27	5.86	[37–39]
Australia	349	25.69	[14, 40–46]
UK	91	67.33	[43, 47–49]
Netherlands & Belgium	137	29.12	[50, 51]
Brazil	27	214.3	[52]
Poland	23	37.75	[53]
Sweden	125	10.42	[54]
Ireland	25	5.03	

The table illustrates the number of individuals with Rett syndrome in relation to the overall population for diverse countries, with references included for data sources. *The numbers of individuals with RTT included in the studies from the specific population mentioned above might have increased, with progressing research exploring a broader population

### Organization and analysis of the data provided by the Irish cohort

Data collected from the Irish Cohort were cross-tabulated, based on the questions asked in the questionnaire. It was then transferred electronically into a database and processed by Microsoft Excel. Numerical data was analysed by calculating the mean of all input provided (age, weight, height, etc.). Non-numerical data was assessed by calculating the percentage of participants who answered ‘yes’ in response to the presence of the feature in question.

### Comparison between data from the Irish RTT population and other cohorts from other countries

A binomial test was used to compare the categorical variables associated with the clinical signs and symptoms displayed by our sample, to populations from other countries. A two-sample *t*-test was carried out to compare the numerical variables associated with the clinical signs and symptoms displayed by our sample, to populations in other countries. This analysis was carried out to assess any significant differences between the cohorts. For all analyses, significance was defined as p ≤ 0.05 adjusted with the Bonferroni correction method.

This is the first study to investigate the Irish RTT population and analyse data from information provided by the caregivers of individuals with RTT. In collaboration with the Rett Syndrome Association of Ireland (RSAI), we prepared a set of questions to collect demographic information and reported clinical presentation. The questionnaires were distributed, collected, and anonymized by the RSAI. The data was subsequently analysed by researchers who were blinded to the participants' identities. In this study, the term “individual” refers to the person diagnosed with Rett syndrome while “participant” refers to the individual’s caregiver. The participants completed the questionnaire, on behalf of the individual in their care. This study focuses on providing a characterization of the RTT population in Ireland and compares it with populations in other countries, with the goal of increasing their global recognition and facilitating collaborative research efforts on an international scale.

## Results

### Analysis of the Rett syndrome demographic data in Ireland

In collaboration with the Rett Syndrome Association of Ireland (RSAI), we prepared a questionnaire based on Grillo and colleagues’ research and distributed it to families in the RSAI community [56]. Twenty-five questionnaires were received for analysis. They are indicative of the trends in symptom presentation seen in the Irish RTT population. To facilitate the analysis, the questionnaire was organized into different sections: general information, diagnosis, regression of acquired skills, presentation of symptoms (gastrointestinal problems, communication, motor skills, epilepsy, breathing abnormalities, cold extremities, skeletal abnormalities, involuntary movements, bruxism), behavior, additional diagnostic testing, family details of RTT individuals in Ireland and pregnancy details.

#### General information

In questions 1–4, general information was collected that is summarized in Table 2. The participants reported that the age at diagnosis was 5.25 ± 6.457 years, and pubertal onset was observed at the age of 11.54 ± 2.241 years. Some participants reported using medication to delay puberty, some reported irregular menstruation, and others reported that the individuals had not yet started menstruating despite experiencing the onset of puberty. Most of the individuals in the study were young adult females.

**Table 2:** General information of individuals with RTT in Ireland

Demographics	Mean ± SD	Range
Current age (years)	20.90 ± 10.855	2 to 46 years
Current height (feet)	4.90 ± 0.440	4 to 5.7 feet
Current weight (kg)	38.00 ± 13.455	10 to 65 kgs
Age of diagnosis (years)	5.25 ± 6.457	1.1 to 30 years

The tabular data show the participants' demographics, including the mean and standard deviation (SD) for their age, height, and weight at the time of the study and age at diagnosis

#### Diagnosis

**Fig. 1:**
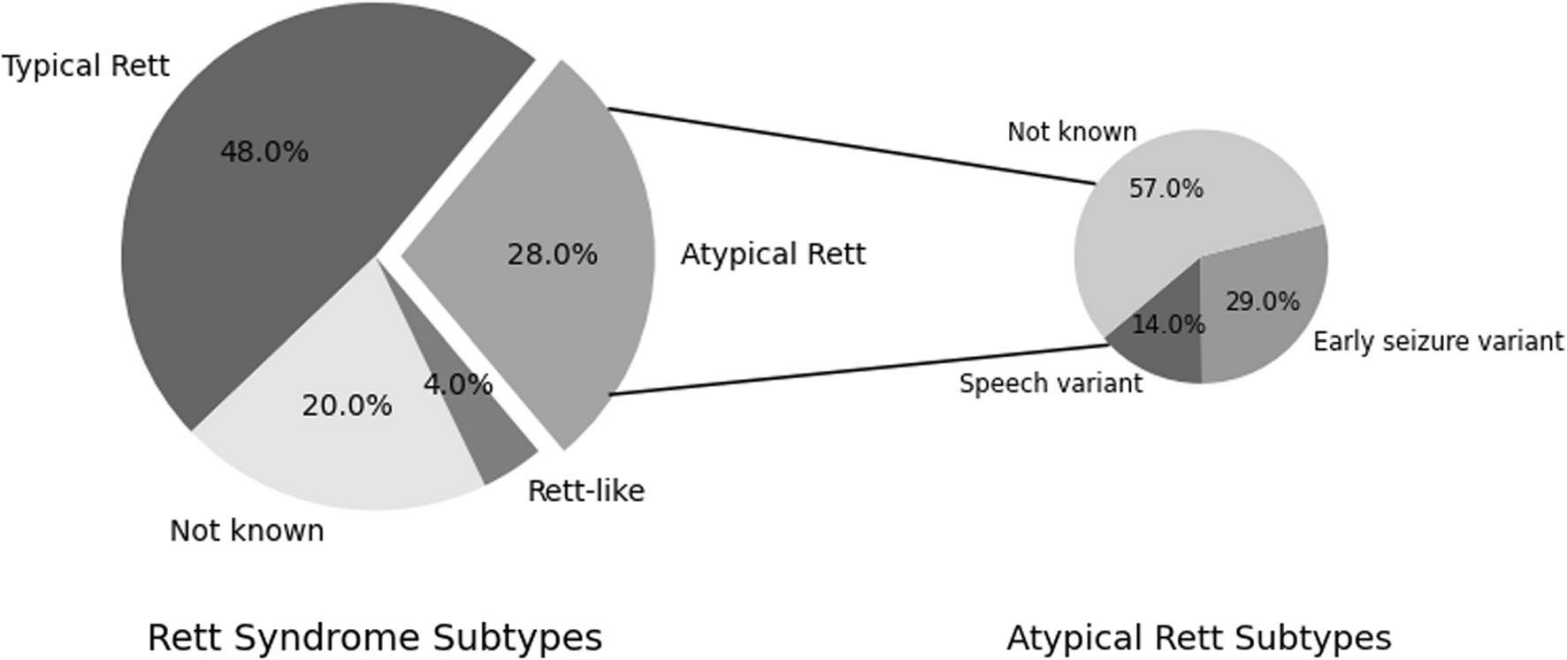
Reported information on the various clinical forms of RTT diagnosed in individuals in Ireland. The left pie chart shows the percentage of different clinical RTT forms- typical, atypical and Rett-like as reported in our study cohort: 48% (n = 12) reported a diagnosis of typical RTT, 28% (n = 7) reported a diagnosis of atypical RTT, 4% (n = 1) reported a ‘Rett-like’ diagnosis, and 20% (n = 5) did not know their diagnosis. The right pie chart shows the different forms of atypical RTT reported by the participants: 14% (n = 1) reported speech variant, 29% (n = 2) reported ‘early seizure variant’, and 57% (n = 4) did not know the atypical RTT variant they were diagnosed with

Questions 5–8 focused on the diagnostic information reported as classic or atypical RTT diagnosis reported in Fig. 1. The study sample consisted of a mix of individuals with reported typical and atypical cases, and some cases lacked a formal diagnosis.

For the information on the type of Rett, 48% (n = 12) reported having a diagnosis of typical RTT and 28% (n = 7) reported having a diagnosis of atypical RTT. 4% (n = 1) of participants reported their RTT subtype as ‘Rett-like’, and 20% (n = 5) did not answer this question. Among the families that reported a diagnosis of atypical RTT, 14% (n = 1) were reported to have speech variant, and 29% (n = 2) were reported to have ‘the early seizure variant’. Genetic test was performed in 88% (n = 22) of individuals, and not performed in 8% (n = 2); and 4% (n = 1) did not answer. Although most of the participants reported that a genetic test was performed, only 40% (n = 10) detailed the mutation identified, and 20% (n = 5) reported general *MECP2* mutations without providing details. 40% (n = 10) did not report any information regarding the mutation. The *MECP2* gene mutations described by the participants were: Point mutations including p.Thr158Met, p.Arg306Cys, p.Pro152Arg, p.Arg133Cys, p.Pro152Arg, Gln128Ter, Cys382Ter; Insertions including C488A > 6; and deletions including C.1162_1172del, c.1164_1207del, other deletions in exons 3 and 4.

Some participants also reported information related to the residence of the individuals. All the individuals with RTT were living in their family home, except one who was reported to live in a residential care facility. Study participants shared details about the hospitals where the individuals were diagnosed. Most individuals were clinically diagnosed in Ireland, although their genetic diagnosis test was carried out in the UK.

#### Regression of acquired skills

Regression (Question 9), a common feature of RTT presentation, was reported based on the six parameters reported in Table 3. For general regression of acquired skills, 96% (n = 24) participants reported ‘yes’, and 4% (n = 1) of participants reported ‘no’. One of the participants reported no observed regression in the individual who was the youngest in the cohort, and it is possible that regression is yet to be observed. 84% (n = 21) of participants reported that regression started at the age of 1.66 ± 0.732 years, while 16% (n = 4) participants did not answer this question. For behavioral regression, 68% (n = 17) participants reported ‘yes’, and 32% (n = 8) participants reported ‘no’. 64% (n = 16) participants reported that behavioral regression started at the age of 1.40 ± 0.415 years, and 36% (n = 9) participants did not answer this question. Regarding speech regression, only 12% (n = 3) of individuals were reported to have the ability to speak using few words, and the remaining 88% (n = 22) of individuals had no ability to speak. In relation to regression of hand use, 88% (n = 22) reported ‘yes’, 8% (n = 2) reported ‘no’, and 4% (n = 1) did not answer. Participants provided information about the individuals’ current hand use: 88% (n = 22) were reported to have loss of normal hand use, 8% (n = 2) had normal hand use, and 4% (n = 1) did not answer. 36% (n = 9) had poor grasp, and in 12% (n = 3) one hand was more functional than the other. A substantial number of participants reported regression in speech, motor skills, and hand use, and there were fewer reports of regression in behavior and growth.

**Table 3:** Regression with their forms observed in individuals with RTT in Ireland

Forms of regression	Frequency	Mean age ± SD (years)	Responses
General regression	96%	1.66 ± 0.732	N = 24
Behaviour regression	68%	1.40 ± 0.415	N = 17
Speech regression	84%	1.60 ± 0.678	N = 21
Motor regression	84%	3.61 ± 4.171	N = 21
Growth failure	48%	4.50 ± 4.855	N = 12
Regression hand use	88%	2.29 ± 1.426	N = 22

The tabular data show the frequency of regression reported by the participants, including general regression, regression in behaviour, speech, motor, growth failure, and hand use. Age (mean and SD) at which specific forms of regression were observed, and the number of responses received for each parameter are mentioned

#### Presentation of symptoms

Questions 10–22 inquire about specific symptoms: GI problems, communication, motor skills, epilepsy and other clinical presentations, and the results are indicated in Table 4.

**Table 4:** Details of symptomatic presentation of the individuals with RTT in Ireland

Problems	Frequency (%)	Responses
**Gastrointestinal issues**		
General gastrointestinal problems	84	N = 21
Constipation	72	N = 18
Reflux	40	N = 10
Chewing and swallowing problems	40	N = 10
PEG fed	16	N = 04
Bloating	52	N = 13
Sphincter control	36	N = 09
**Communication**		
Verbal communication	16	N = 04
Nonverbal communication	80	N = 20
Ability to understand & interact	68	N = 17
**Motor skills**		
Ability to sit	56	N = 14
Ability to walk	20	N = 05
Ability to stand	52	N = 13
Walking with support	52	N = 13
**Epilepsy**		
Current medications	88	N = 22
Seizures	76	N = 19
Resistance to medications	12	N = 03
Family history of seizures	12	N = 03
**Other clinical presentations**		
Breathing abnormalities	64	N = 16
Cold extremities	84	N = 21
Skeletal abnormalities	60	N = 15
Involuntary movements	84	N = 21
Bruxism	56	N = 14

The tabular data show the frequency of issues such as gastrointestinal, motor, additional clinical presentation, communication, and epilepsy; the number of responses received for each parameter are mentioned

##### Gastrointestinal problems

Gastrointestinal issues (Question 11) were reported by 84% (n = 21) of the participants and 16% (n = 4) did not report them. Regarding the specific GI problems, constipation was reported in 72% (n = 18) and bloating in 52% (n = 13) of the individuals. 36% (n = 9) of the individuals could control their sphincter muscle, 44% (n = 11) could not, and 20% (n = 5) of the participants did not answer. Reflux was reported in 40% (n = 10) of the individuals, chewing and swallowing difficulties in 40% (n = 10), and 16% (n = 4) of the individuals were fed through a percutaneous endoscopic gastrostomy (PEG) feeding tube (Table 4). Constipation and bloating were the most common GI problems reported by the participants; other issues such as reflux, chewing difficulties, and swallowing problems were less common.

##### Communication

In relation to communication (Question 12), 80% (n = 20) of participants reported that individuals predominantly engaged in nonverbal forms of communication, while 16% (n = 4) indicated that individuals utilized verbal communication, and 12% (n = 3) reported no form of communication (Table 4). Primarily, individuals employed nonverbal methods to communicate, with eye contact comprising 68% (n = 17) of the reported behaviours. 28% (n = 7) employed alternative forms of communication, such as babble speech, mobilization, attention-seeking through vocalizations, head gestures to convey 'no,' displaying agitated eye contact during stress, vocalizing distress or pain, and modulating responses depending on the context. Among this study group, 4% (n = 1) retained full speech, and 8% (n = 2) exhibited limited speech. The individual with full speech had the preserved speech variant and those with limited speech were associated with either the early seizure variant or the classical RTT. Notably, one participant did not specify the RTT subtype for the individual with limited speech. The results from this section indicate that a high percentage of the individuals in the study, 68% (n = 17), maintained the ability to understand and interact with others, while it was unclear in 16% (n = 4) of the individuals. In general, participants observed that individuals primarily relied on nonverbal communication, particularly through eye contact or gazing.

##### Motor skills

The motor skills of individuals (Question 13) were categorized based on their abilities to sit, stand, walk with support, and walk without support. The resulting data from these questions are represented in Table 4. 16% (n = 4) of the participants reported the individual’s ability to stand with support, and 5% (n = 1) needed support with walking during periods of regression. In 4% (n = 1), the individual’s ability to walk/stand was lost at ~ 5 years of age. Approximately half of the individuals could stand, sit, and walk with assistance. Only 20% (n = 5) were able to walk independently.

##### Epilepsy

**Fig. 2:**
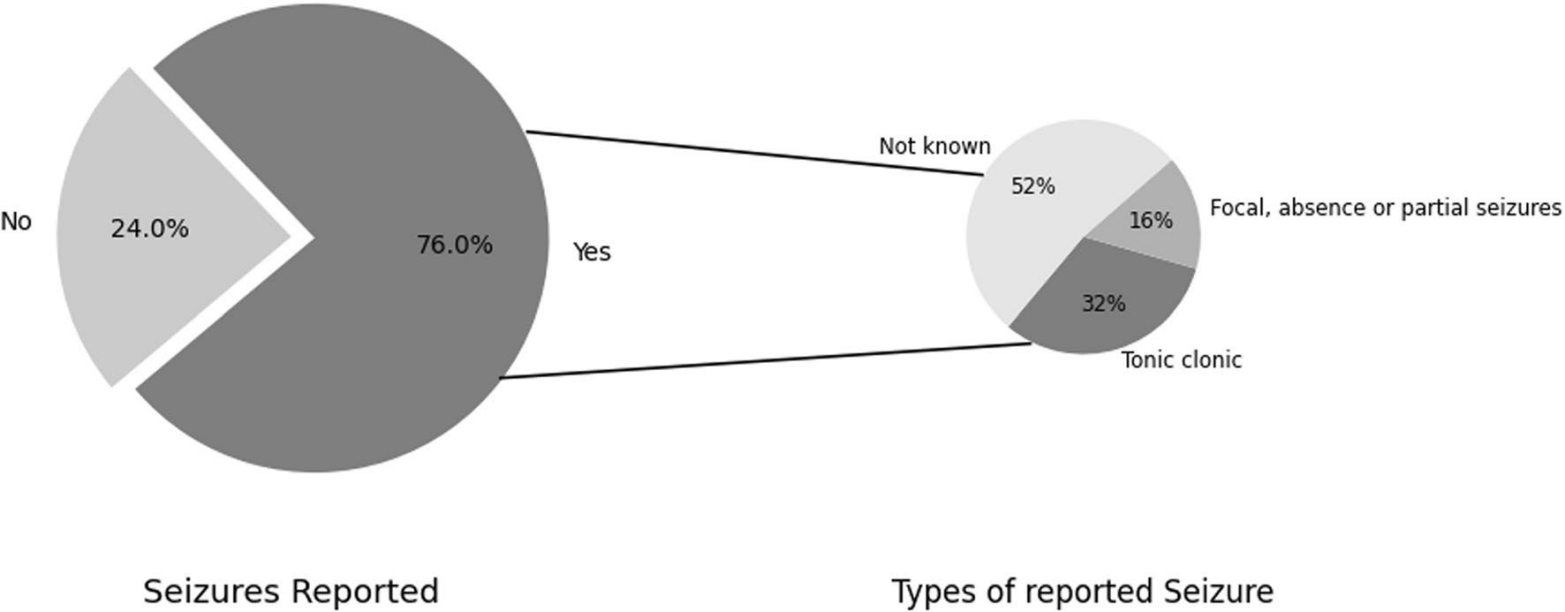
Reported information on the frequency and forms of seizures reported in individuals in Ireland. The larger pie chart shows the percentage of seizures as reported in our study cohort: 76% (n = 19) reported experiencing epileptic seizures, while 24% (n = 6) did not. The smaller pie chart shows the different forms of seizures reported by the participants: 32% (n = 6) exhibited tonic clonic seizures, 16% (n = 3) exhibited focal, absence or partial seizures, and the remaining 52% (n = 10) did not know the type of seizure they experienced

The epileptic status (Question 14) of individuals was enquired in relation to the parameters exhibited in Table 4. 76% (n = 19) of the participants reported that the individuals experienced epileptic seizures from the average age of 6.72 ± 3.735 years. Participants reported diverse seizure frequencies and characteristics. Seizures were described in detail by 48% (n = 9) of participants who experienced them and reported that 32% (n = 6) of individuals exhibited tonic–clonic seizures, and 16% (n = 3) exhibited focal, absence, or partial seizures (Fig. 2). One individual experienced solely facial seizures, and another had seizures exclusively during sleep. Uncertainty arose regarding the nature of epilepsy in the response of one participant, which was described as "true epilepsy" or a "Rett-event" by their medical consultant. Seizures were reportedly controlled or had ceased in two individuals when reported during the data collection of this study, and for one individual, the onset of epilepsy coincided with the start of menstruation.

The participants reported that 88% (n = 22) of the individuals were taking medications for epilepsy and 12% (n = 3) had not responded to anti-epileptic drugs. A family history of temporal lobe epilepsy was reported in 4% (n = 1), and an unspecified family history of epilepsy was reported in 8% (n = 2) of the individuals. In summary, a notable subset of individuals was reported to have seizures. Most of the individuals were prescribed anti-epileptic medications, and only a small subset was reported to exhibit limited responsiveness to the administered pharmacological interventions.

##### Breathing abnormalities

Participants reported breathing abnormalities (Question 15) in 64% (n = 16) of the individuals, which ranged from irregular patterns of rapid breathing, breath holding, hyperventilation, shallow breathing, and apnea.

##### Cold extremities

One of the common symptoms of RTT is cold extremities; this was reported by 84% (n = 21) of participants. In some individuals, only the feet were affected.

##### Skeletal abnormalities

60% (n = 15) of the individuals were reported to exhibit skeletal abnormalities (Question 19) including scoliosis. These abnormalities were reported to cause pain and discomfort affecting the individual’s ability to walk and move. There were no reported skeletal abnormalities in 36% (n = 9) of the individuals. 4% (n = 1) of the participants did not respond to this question. Two individuals had undergone scoliosis surgery to address their condition.

##### Involuntary movements

Involuntary movements (Question 20) were reported in 84% (n = 21) of the individuals. These included hand wringing, repetitive finger movements, and irregular movements of the limbs and trunk. There were no reports of involuntary movement in 12% (n = 3) of the individuals, and 4% (n = 1) of the participants did not answer the question.

##### Bruxism

Bruxism (Question 21), another common feature of RTT, was reported in 56% (n = 14) of the individuals while 28% (n = 7) did not present bruxism and 16% (n = 4) of the participants did not answer the question.

To summarize, stereotypic involuntary movements and cold extremities were commonly reported by most participants, while breathing issues, skeletal problems, and bruxism were found to be less prevalent, as summarized in Table 4. Additional symptoms including eosinophilic esophagitis, sinus congestion, gum disease, and gastric problems were reported in some individuals.

#### Behaviour

**Fig. 3:**
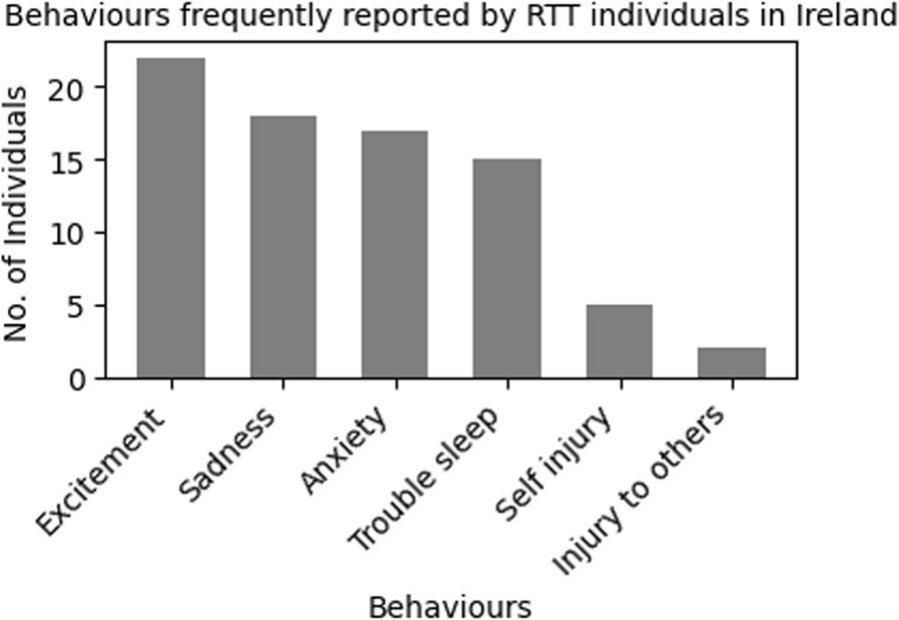
Details of behavioural presentation of the individuals with RTT in Ireland. The bar chart shows the number of individuals displaying behaviours, relating to excitement, sadness, anxiety, difficulty sleeping during night, self-injury, and injury to others

The behaviours most frequently observed in our cohort are represented in Fig. 3. The most reported behavior (Question 23) was excitement in 92% (n = 22), sadness in 75% (n = 18), anxiety in 71% (n = 17), night-time sleep disturbance in 63% (n = 15), self-injury in 21% (n = 5) and injury to others in 8% (n = 2).

#### Additional diagnostic testing

**Fig. 4:**
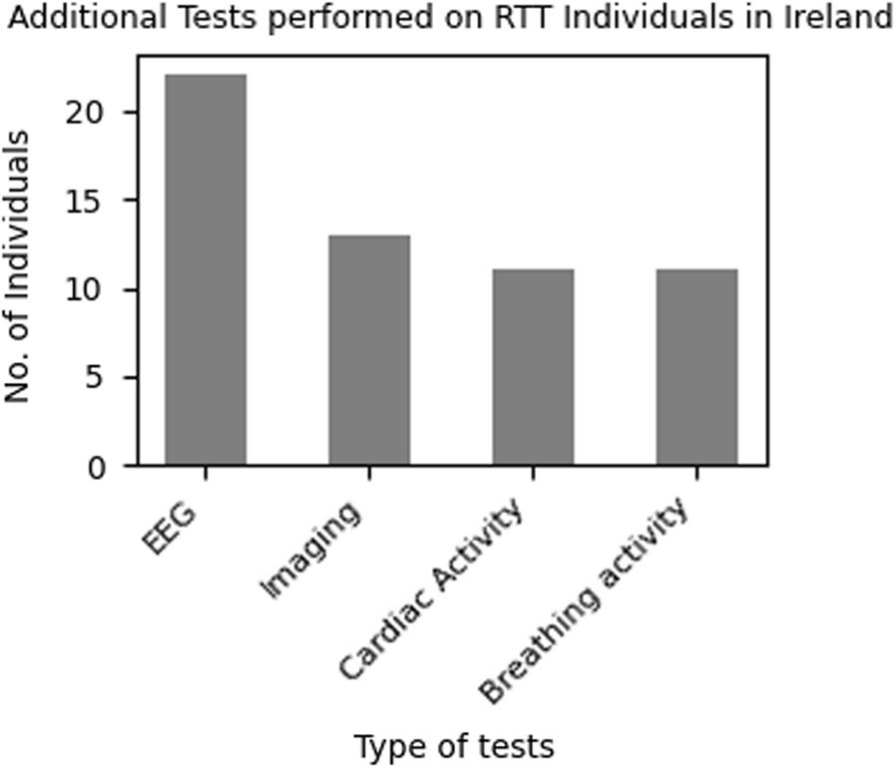
Details of diagnostic tests performed on the individuals with RTT in Ireland. The bar chart shows the number of individuals who underwent additional diagnostic tests including EEG, cardiac activity, breathing activity and imaging

Figure 4 illustrates the distribution of the individuals who underwent additional diagnostic tests as reported by the participants (Question 24) in our study. Electroencephalogram (EEG) tests were conducted in 88% (n = 22) of the individuals, imaging tests were performed in 52% (n = 13), and evaluation of breathing and cardiac activity was performed in 44% (n = 11) of the individuals. The data pertaining to these tests was derived from responses provided by caregivers of individuals with Rett syndrome who indicated "yes" when asked about the tests, if conducted. However, specific scan details necessary for drawing more definitive results were not available. The most frequently performed test was EEG, while assessments related to breathing activity, cardiac activity, and imaging were less frequently conducted.

#### Family details of individuals with RTT in Ireland

The participants were questioned about family data (Question 25) such as the presence of consanguinity (between parents), a family history of RTT, other diseases present in the family, and the number, age, and sex of siblings. A family history of other diseases including heart disease, thyroid disease, depression, anxiety, psoriasis, psoriatic arthritis, multiple sclerosis, autism, and type II diabetes was reported by 28% (n = 7) of the participants. 88% (n = 22) of participants reported that the individuals had siblings, of whom 76% (n = 19) shared information about the sex of the individual’s siblings. The mean age of the individual’s siblings was 20.82 years. There was no family history of RTT reported. Consanguinity between parents of the individual was reported by 4% (n = 1) of participants. Approximately half of the participants reported a family history of various other disorders but not RTT.

#### Details of pregnancy

4% (n = 1) of the participants reported multiple pregnancies when describing the details of pregnancy (Question 26). 12% (n = 3) of the mothers had taken medications, including carbamazepine and heparin, during pregnancy. Assisted delivery interventions were reported in 44% (n = 10) of the cases; caesarean deliveries accounted for 80% of these interventions. Other complications reported were the use of forceps, face presentation, intrauterine blood transfusion, postnatal blood transfusion, and obstructed labour.

### Analysis of the population data of individuals with RTT in Ireland and comparison with other countries

#### Representation of parameters in populations with RTT across several countries

The following section explores a comparison of a wide range of parameters across several RTT populations. Factors including individual’s age, weight, diagnosis age, regression observational age, and seizure onset were selected to compare Ireland and several other countries. The frequency of RTT characteristics such as general regression, behavioral regression, speech, motor skills, hand use, stereotypies, gastrointestinal issues, communication including verbal and nonverbal, eye communication, the individual’s ability to sit, stand and walk (with and without support), epilepsy (seizures), resistance to anti-epileptic drugs, breathing abnormalities, bloating, cold extremities, sphincter control, skeletal abnormalities, involuntary movements, bruxism, behaviours such as anxiety, self-injury, trouble night sleep, and sadness are also included in this analysis. The findings are organized in Tables 5 and 6 for each population's data. The literature available to date does not include data for all variables across all populations [4, 14, 30–55]. Notably, a recent study by Neul et al., focused on determining the longitudinal distribution of hand function skills in individuals with classic Rett syndrome in the USA [58].

**Table 5:** Frequency of RTT characteristics in the Irish population compared to populations in other countries

Characteristic	Percentage of individuals with characteristic										
	US	IT	DK	NL	SE	AU	UK	BR	PL	INTL	IE
Regression	90	–	–	–	–	76	95.6	–	–	80	**96** Δ
Behavioural regression	–	–	–	–	–	–	77	–	–	–	**68** ∇
Speech regression	87	50	–	–	23	–	–	–	–	–	84
Motor regression	55	–	–	–	–	–	–	–	–	–	**84** Δ
Regression of hand use	–	–	41	41	–	–	–	–	–	–	**88** Δ
Stereotypies	86	98*	–	96	–	92	99**	–	70	69	84
GI problems	92	89	–	–	–	83	82	81	–	40	84
Verbal communication	–	16	–	13	–	5	21	–	–	–	16
Non-verbal communication	–	76	–	86	65	39	79	–	–	70	80
Eye communication	–	–	85	–	–	92	91	–	–	89	85
Ability to sit	–	37	85**	–	–	–	–	59	–	–	56
Ability to walk	–	20	44*	33	80**	56**	66**	22	–	44*	20
Ability to stand	–	–	–	–	–	46	28	–	–	–	**52** Δ
Walking with support	–	41	41	–	–	43	64	11	–	–	52
Seizures	81	82	80	61	–	81	63	69	43	65	76
Resistance to anti-epileptics	–	36*	46**	18	–	36*	–	–	–	25	**12** ∇
Breathing abnormalities	–	70	75	81*	85*	–	66	–	87*	51	64
Bloating	–	–	–	–	–	53	–	–	–	–	52
Cold extremities	–	87	–	56	93	–	–	–	–	50	84
Sphincter control	–	–	–	–	–	–	30	–	–	89	36
Skeletal abnormalities	27	56	83*	70	74	66	67	59	13	50	60
Involuntary movements	–	–	–	40	–	–	–	–	–	–	**84** Δ
Bruxism	–	–	–	–	–	–	–	–	22	61	56
Anxiety	14	–	–	–	–	79	74	–	–	–	71
Self-injury	–	–	–	–	–	62**	57**	–	–	–	**21** ∇
Trouble night sleep	–	77	–	16	–	63	46	–	–	47	63
Sadness	–	–	–	12	–	–	–	–	–	–	**75** Δ

The tabular representation of RTT characteristics in the study cohort in Ireland and other international cohorts, from the USA, Italy, Denmark, the Netherlands, Sweden, Australia, the UK, Brazil, Poland, and an international cohort. The numbers in the figure are accompanied by asterisks to indicate the level of significance of the differences from Ireland (IE). A single asterisk (*) indicates a significant difference from Ireland for the indicated distinctive symptom. The double asterisks (**) indicate a higher level of significance, determined following p-value adjustment for multiple testing. The numbers shown in bold indicate a higher and lower frequency of occurrence reported in Ireland when compared to reported data from other countries. This table provides a visual representation of the differences in the frequency of occurrence of distinctive symptoms between the cohort in Ireland and highlights the significant differences and extreme end frequencies. Instances where the frequency of findings in Ireland is the highest among all other countries are denoted by the symbol "Δ", and when the frequency of findings in Ireland is the lowest, it is denoted by the symbol “∇”

**Table 6:** Representation of the data in the RTT cohort of Ireland and other countries

Characteristic	Mean value within RTT population										
	US	IT	DK	NL	SE	AU	UK	BR	PL	INTL	IE
Sample size	919	84	27	137	125	349	91	27	23	–	25
Age (years)	3**	29**	40.7**	14.9*	19.6	13.7**	20.5	12.2**	7.22**	–	20.9
Current weight (kg)	–	–	42	–	–	–	–	–	–	–	38
Age at diagnosis (years)	2.5**	–	2*	7.8	–	5.5	3	–	3.5	–	5.25
Age at regression (years)	–	–	–	1**	–	2.6**	1.5	–	–	–	1.66
Age at seizure onset (years)	–	5*	–	4	–	3.4**	–	–	–	–	**6.72** Δ

The tabular representation of data such as the current age and weight of the individuals, age of diagnosis, age of regression observed, and age of onset of seizures in the study cohort in Ireland and other international cohorts, such as the USA, Italy, Denmark, Netherlands, Australia, UK, Brazil, and Poland. The ratio of individuals with RTT included in studies from specific countries relative to their overall populations is reported in the methods. The numbers in the figure are accompanied by asterisks to indicate the level of significance of the differences from Ireland (IE). A single asterisk (*) indicates a significant difference from Ireland for the indicated distinctive parameter. The double asterisks (**) indicate a higher level of significance, determined following p-value adjustments for multiple testing. The numbers shown in bold for Ireland indicate an extreme end parameter that has been reported in Ireland and differs greatly from other countries. Instances where the frequency of findings in Ireland is the highest among all other countries are denoted by the symbol "Δ"

#### Frequency of different RTT forms in different populations

The distribution of different RTT types in several population datasets, including those from the international cohort, United States, Italy, the Netherlands, Australia, the United Kingdom, Brazil, and Ireland, is shown in Table 7. The prevalence of RTT, which ranges between 65- 85% for classical RTT and 13–35% for atypical RTT, is consistent in Ireland for the atypical forms, but not for the classical RTT.

**Table 7:** Distribution of RTT forms in Ireland compared to individuals in the other countries

RTT type	RTT types and percentage distribution of affected individuals										
	US	IT	DK	NL	SE	AU	UK	BR	PL	INTL	IE
Typical RTT	85	65	–	80	–	68	76	68	–	80	**48**∇
Atypical RTT	13	35	–	20	–	32	21	27	–	20	28

The tabular representation of RTT forms- classic/typical and atypical RTT in the study cohort in Ireland and other international cohorts, from the USA, Italy, Denmark, the Netherlands, Sweden, Australia, the UK, Brazil, Poland, and an international cohort. The figures in Ireland could be affected by the lack of diagnosis and genetic information. The numbers shown in bold for Ireland indicate an extreme end parameter that has been reported in Ireland and differs greatly from other countries. Instances where the frequency of findings in Ireland is the lowest among all other countries, it is denoted by the symbol “∇”

## Discussion

This is the first study in Ireland that has collated demographic data and clinical characteristics from individuals diagnosed with RTT. In collaboration with the RSAI, we presented a questionnaire to the caregivers to gather information about the clinical and behavioral presentation of Irish individuals with RTT. The results indicate that RTT individuals in Ireland display an overall presentation that is comparable with other countries. Certain characteristics in the Irish cohort appear to be less severe such as breathing problems, resistance to anti-epileptic drugs (AEDs), and self-injury tendencies, however, an accurate genetic diagnosis would sharpen their clinical characterization. The study reveals the genetic diagnosis in a significant number of individuals is unknown/absent, due to limited access to genotyping facilities and diagnostic services available publicly in Ireland. This may lead to possible misdiagnosis of atypical RTT cases [59]. Numerous individuals underwent genetic testing for diagnosis, yet a significant number remain unaware of the specific mutations affecting them. Genetic information is mostly acquired outside of Ireland and relayed to the attending clinician, who subsequently communicates it to the family. Unfortunately, in many instances, the family does not maintain records. Research studies, like the one presented here, or natural history studies, play a crucial role in consolidating clinical, behavioral, and molecular data to enhance the characterization of the population. Notably, the healthcare system in Ireland is progressing towards proactive record-keeping, promising immediate benefits for patients and an overall improvement in the situation.

According to the global prevalence of RTT (5–10 per 100,000 females), one would expect approximately 180 individuals with RTT in Ireland. However, the RSAI reports involvement from 75 families. The sample size in this study may be attributed to limited enrollment with RSAI or the possibility of a misdiagnosis, especially for older individuals. Nevertheless, the ratio of individuals in this study compared to the total population in Ireland is 1 in 200,000, a figure closely aligned with ratios reported in other populations [4, 14, 36–55]. The retained information, including the personal data of individuals with RSAI, can serve as a registry for individuals in Ireland. This registry could then be integrated into or contribute to other registries. This research includes both typical and atypical cases of RTT, with 28% of the participants reporting the atypical variant, however, this could be affected by the limited genetic information. All participants reported shorter stature in relation to the individual’s weight and age, aligning with prior literature that highlights the likelihood of short stature among females with a *MECP2* mutation [31]. This observation agrees with the pattern of deficits in height-for-age and weight-for-height ratios previously documented [31, 52, 60]. A small percentage of participants reported that individuals had excess weight for age and height, similar to children with autism spectrum disorder [61]. The average age of RTT diagnosis in Ireland is 5.25 years, which is higher than that in the Poland [53], the UK [43, 47–49], the USA and Canada [30–35], and Denmark [37–39], but lower than that in Australia [7, 40–46], and the Netherlands and Belgium. The present study confirms that clinical severity worsens with age, and that there is a correlation between delayed onset of RTT and preserved hand function. Despite limited access to RTT genetic diagnosis in Ireland, the average age of diagnosis seems to be comparable to that of other populations [7, 30]. Nonetheless, these results are valuable, considering the proportion of Rett individuals in studies from other countries relative to their populations aligns closely with the situation observed in Ireland. They provide insight into the phenotypic presentation of individuals with RTT in Ireland and prompt to action in providing a better characterization of the population and a natural history study.

RTT affects growth and is associated with issues such as feeding difficulties, motor function, digestion, apraxia, hyperventilation, sleep, and scoliosis [62]. Our findings indicate a progression of behavioral challenges with advancing age, as older individuals tend to exhibit increased agitation, while younger individuals exhibit more positive moods, in line with previous literature [63]. Constipation is a commonly reported gastrointestinal issue. Some individuals are PEG-fed or have a gastrostomy to meet their nutritional needs, which helps maintain their BMI [64]. Certain participants in our study in the age range of 15–39 years reported the absence of bruxism, in accordance with earlier investigations indicating a diminishing prevalence of bruxism with advancing age [65]. Differences in participant characteristics, particularly their age, contribute to variations when comparing findings with reports from other countries. For example, Denmark stands out with the oldest average age of RTT individuals among the study populations, reporting in a higher prevalence of skeletal abnormalities, improved sitting ability, and increased resistance to anti-epileptic drugs. In contrast, the USA features the youngest average age of RTT individuals among the studied populations, reporting higher incidence of GI issues, more frequent seizures, a lower occurrence of skeletal abnormalities, and a reduced frequency of anxiety in comparison to other populations [4, 14, 36–55]. Although some of the parameters are comparable to other cohorts, RTT individuals in Ireland display late onset of seizures, marked regression and involuntary movements. Most participants reported individuals taking medications for epilepsy, with a minority reported to be resistant to anti-epileptics. These diverse findings underscore the heterogeneity of seizure presentations in Rett syndrome and emphasize the importance of individualized approaches to diagnosis and management. Nutritional interventions such as correcting vitamin D deficiency can permit lower anti-epileptic drug dosages and improve health [66, 67]. Low levels of Vitamin D in RTT individuals have been previously reported to be more prominent in summer than in winter and can be related to less sun exposure due to restricted mobility and the necessity for assistance, prompting prolonged indoor stays. Vitamin D deficiency in Ireland impacts approximately one in four children, showing a higher prevalence among females and those aged over 12 years [67, 68]. Skeletal abnormalities and bone density can depend on vitamin D deficiencies. Preventive measures may involve increasing sunlight exposure without sunscreen for natural synthesis and incorporating vitamin D fortified beverages or multivitamin supplements, resulting in improved vitamin D status in these individuals [69]. Considering the number of individuals with RTT presenting with this deficiency, monitoring of bone density would be recommended. The literature confirms our finding that cold extremities are more prevalent in Sweden than in Ireland due to the country's cooler climate [67]. None of the individuals in our study had a family history of RTT and consanguinity between parents, was only reported for one individual. Nearly half of the participants reported that the individuals were delivered via C-sections, which has been reported to increase the risk of neurodevelopmental conditions [70].

Research indicates that Irish individuals with Rett syndrome are not currently participating in worldwide studies or clinical trials. To address this issue, a natural history of Irish RTT population would benefit the characterization of the individuals and their participation in international studies. We plan to develop a database that will consist of clinical details relating to RTT individuals in Ireland, in compliance with GDPR regulations and ethical standards. Our goal is to increase the visibility of the Irish population with RTT on a global platform and to encourage future research and foster collaboration. Multiple existing centralized RTT databases have contributed to improving diagnosis, treatment, and future developments for RTT individuals. The inclusion of Irish individuals in these databases would be beneficial for RTT individuals, researchers, healthcare professionals and the Irish community.

## Conclusion

We have provided an overview of the demographic and clinical features of individuals with Rett syndrome (RTT) in Ireland, marking this study as the first of its kind in the country. Collaborating with the RSAI, we presented a questionnaire to caregivers aimed at gathering the clinical and behavioral presentation of individuals with Rett in Ireland. The average age of RTT diagnosis in Ireland, at 5.25 years, is comparable to that reported in other populations. Our findings suggest that individuals with RTT in Ireland generally exhibit clinical presentations comparable with those observed in other countries. Interestingly, certain symptoms among the Irish cohort appear less severe, including reduced incidence of breathing difficulties, resistance to anti-epileptic drugs (AEDs), and self-injurious tendencies. It's worth noting that a substantial number of individuals in our study lack genetic diagnosis due to limited access to genotyping facilities available in Ireland. However, the accuracy of genetic diagnosis emerges as a crucial factor that could further refine their clinical characterization. The present study highlights an essential concern: Irish individuals with RTT are currently not actively participating in global studies or clinical trials. Our study aims to elevate the visibility of the Irish RTT population on the global stage, fostering future research and international collaboration.

## Data Availability

The datasets generated and/or analysed during the current study are not publicly available due to individual patient privacy and lack of consent but are available from the corresponding author on reasonable request.

## Abbreviations

ARSD: Australian Rett Syndrome Database
EEG: Electroencephalogram
FDA: Food and Drug Administration
GI: Gastrointestinal
MECP2: Methyl-CpG binding protein 2
miRNA: MicroRNA
RSAI: Rett Syndrome Association of Ireland
RTT: Rett syndrome
Z-RTT: Zapella Rett syndrome variant

## References

[1] Rett A (1966). On a unusual brain atrophy syndrome in hyperammonemia in childhood. Wien Med Wochenschr.

[2] Petriti U, Dudman DC, Scosyrev E, Lopez-Leon S (2023). Global prevalence of Rett syndrome: systematic review and meta-analysis. Syst Rev.

[3] Hagberg B (2002). Clinical manifestations and stages of Rett syndrome. Ment Retard Dev Disabil Res Rev.

[4] Pini G, Bigoni S, Congiu L, Romanelli AM, Scusa MF, Di Marco P (2016). Rett syndrome: a wide clinical and autonomic picture. Orphanet J Rare Dis.

[5] Naidu S, Bibat G, Kratz L, Kelley RI, Pevsner J, Hoffman E (2003). Clinical variability in Rett syndrome. J Child Neurol.

[6] Neul JL (2012). The relationship of Rett syndrome and MECP2 disorders to autism. Dialogues Clin Neurosci.

[7] Fehr S, Bebbington A, Nassar N, Downs J, Ronen GM, De Klerk N (2011). Trends in the diagnosis of Rett syndrome in Australia. Pediatr Res.

[8] Neul JL, Benke TA, Marsh ED, Skinner SA, Merritt J, Lieberman DN (2019). The array of clinical phenotypes of males with mutations in Methyl-CpG binding protein 2. Am J Med Genet B Neuropsychiatr Genet.

[9] Hagberg B, Aicardi J, Dias K, Ramos O (1983). A progressive syndrome of autism, dementia, ataxia, and loss of purposeful hand use in girls: Rett's syndrome: report of 35 cases. Ann Neurol.

[10] Hagberg B, Hanefeld F, Percy A, Skjeldal O. An update on clinically applicable diagnostic criteria in Rett syndrome. Comments to Rett Syndrome Clinical Criteria Consensus Panel Satellite to European Paediatric Neurology Society Meeting, Baden Baden, Germany, 11 September 2001. Eur J Paediatr Neurol. 2002;6(5):293–7. 10.1053/ejpn.2002.0612 12378695

[11] Hagberg BA, Skjeldal OH (1994). Rett variants: a suggested model for inclusion criteria. Pediatr Neurol.

[12] Neul JL, Kaufmann WE, Glaze DG, Christodoulou J, Clarke AJ, Bahi-Buisson N (2010). Rett syndrome: revised diagnostic criteria and nomenclature. Ann Neurol.

[13] Schonewolf-Greulich B, Bisgaard AM, Moller RS, Duno M, Brondum-Nielsen K, Kaur S (2019). Clinician's guide to genes associated with Rett-like phenotypes-Investigation of a Danish cohort and review of the literature. Clin Genet.

[14] Amir RE, Van den Veyver IB, Wan M, Tran CQ, Francke U, Zoghbi HY (1999). Rett syndrome is caused by mutations in X-linked MECP2, encoding methyl-CpG-binding protein 2. Nat Genet.

[15] Ross PD, Guy J, Selfridge J, Kamal B, Bahey N, Tanner KE (2016). Exclusive expression of MeCP2 in the nervous system distinguishes between brain and peripheral Rett syndrome-like phenotypes. Hum Mol Genet.

[16] Ip JPK, Mellios N, Sur M (2018). Rett syndrome: insights into genetic, molecular and circuit mechanisms. Nat Rev Neurosci.

[17] Suter B, Treadwell-Deering D, Zoghbi HY, Glaze DG, Neul JL (2014). Brief report: MECP2 mutations in people without Rett syndrome. J Autism Dev Disord.

[18] Percy AK, Neul JL, Glaze DG, Motil KJ, Skinner SA, Khwaja O (2010). Rett syndrome diagnostic criteria: lessons from the Natural History Study. Ann Neurol.

[19] Banerjee A, Miller MT, Li K, Sur M, Kaufmann WE (2019). Towards a better diagnosis and treatment of Rett syndrome: a model synaptic disorder. Brain.

[20] Gold WA, Krishnarajy R, Ellaway C, Christodoulou J (2018). Rett syndrome: a genetic update and clinical review focusing on comorbidities. ACS Chem Neurosci.

[21] Percy AK, Lane J, Annese F, Warren H, Skinner SA, Neul JL (2018). When Rett syndrome is due to genes other than MECP2. Transl Sci Rare Dis.

[22] Iwama K, Mizuguchi T, Takeshita E, Nakagawa E, Okazaki T, Nomura Y (2019). Genetic landscape of Rett syndrome-like phenotypes revealed by whole exome sequencing. J Med Genet.

[23] Cuddapah VA, Pillai RB, Shekar KV, Lane JB, Motil KJ, Skinner SA (2014). Methyl-CpG-binding protein 2 (MECP2) mutation type is associated with disease severity in Rett syndrome. J Med Genet.

[24] Bebbington A, Downs J, Percy A, Pineda M, Zeev BB, Bahi-Buisson N (2012). The phenotype associated with a large deletion on MECP2. Eur J Hum Genet.

[25] Colvin L, Leonard H, de Klerk N, Davis M, Weaving L, Williamson S (2004). Refining the phenotype of common mutations in Rett syndrome. J Med Genet.

[26] Neul JL, Fang P, Barrish J, Lane J, Caeg EB, Smith EO (2008). Specific mutations in methyl-CpG-binding protein 2 confer different severity in Rett syndrome. Neurology.

[27] Knudsen GPS, Neilson TCS, Pedersen J, Kerr A, Schwartz M, Hulten M (2006). Increased skewing of X chromosome inactivation in Rett syndrome patients and their mothers. Eur J Hum Genet.

[28] Zhang Q, Zhao Y, Bao X, Luo J, Zhang X, Li J (2017). Familial cases and male cases with MECP2 mutations. Am J Med Genet B Neuropsychiatr Genet.

[29] Fang X, Butler KM, Abidi F, Gass J, Beisang A, Feyma T (2022). Analysis of X-inactivation status in a Rett syndrome natural history study cohort. Mol Genet Genomic Med.

[30] Tarquinio DC, Hou W, Neul JL, Lane JB, Barnes KV, O'Leary HM, et al. Age of diagnosis in Rett syndrome: patterns of recognition among diagnosticians and risk factors for late diagnosis. Pediatr Neurol. 2015;52(6):585–91 e2. 10.1016/j.pediatrneurol.2015.02.007 PMC4442062 25801175

[31] Motil KJ, Caeg E, Barrish JO, Geerts S, Lane JB, Percy AK (2012). Gastrointestinal and nutritional problems occur frequently throughout life in girls and women with Rett syndrome. J Pediatr Gastroenterol Nutr.

[32] Percy AK. Progress in Rett Syndrome: from discovery to clinical trials. Wiener medizinische Wochenschrift (1946). 2016;166(11–12):325–32. 10.1007/s10354-016-0491-9 PMC5005392 27491553

[33] Kirby RS, Lane JB, Childers J, Skinner SA, Annese F, Barrish JO, et al. Longevity in Rett syndrome: analysis of the North American Database. J Pediatr. 2010;156(1):135–8 e1. 10.1016/j.jpeds.2009.07.015 PMC2794941 19772971

[34] Buchanan CB, Stallworth JL, Joy AE, Dixon RE, Scott AE, Beisang AA (2022). Anxiety-like behavior and anxiolytic treatment in the Rett syndrome natural history study. J Neurodev Disord.

[35] Killian JT, Lane JB, Lee HS, Skinner SA, Kaufmann WE, Glaze DG (2017). Scoliosis in Rett Syndrome: progression, comorbidities, and predictors. Pediatr Neurol.

[36] Vignoli A, La Briola F, Peron A, Turner K, Savini M, Cogliati F (2012). Medical care of adolescents and women with Rett syndrome: an Italian study. Am J Med Genet A.

[37] Schonewolf-Greulich B, Stahlhut M, Larsen JL, Syhler B, Bisgaard AM (2017). Functional abilities in aging women with Rett syndrome—the Danish cohort. Disabil Rehabil.

[38] Bisgaard AM, Wong K, Hojfeldt AK, Larsen JL, Schonewolf-Greulich B, Ronde G (2021). Decline in gross motor skills in adult Rett syndrome; results from a Danish longitudinal study. Am J Med Genet A.

[39] Marschik PB, Lemcke S, Einspieler C, Zhang D, Bolte S, Townend GS (2018). Early development in Rett syndrome—the benefits and difficulties of a birth cohort approach. Dev Neurorehabil.

[40] Laurvick CL, de Klerk N, Bower C, Christodoulou J, Ravine D, Ellaway C (2006). Rett syndrome in Australia: a review of the epidemiology. J Pediatr.

[41] Foley KR, Downs J, Bebbington A, Jacoby P, Girdler S, Kaufmann WE (2011). Change in gross motor abilities of girls and women with rett syndrome over a 3- to 4-year period. J Child Neurol.

[42] Colvin L, Fyfe S, Leonard S, Schiavello T, Ellaway C, De Klerk N (2003). Describing the phenotype in Rett syndrome using a population database. Arch Dis Child.

[43] Robertson L, Hall SE, Jacoby P, Ellaway C, de Klerk N, Leonard H (2006). The association between behavior and genotype in Rett syndrome using the Australian Rett Syndrome Database. Am J Med Genet B Neuropsychiatr Genet.

[44] Anderson A, Wong K, Jacoby P, Downs J, Leonard H (2014). Twenty years of surveillance in Rett syndrome: What does this tell us?. Orphanet J Rare Dis.

[45] Downs J, Torode I, Wong K, Ellaway C, Elliott EJ, Christodoulou J, et al. The natural history of scoliosis in females with Rett syndrome. Spine (Phila Pa 1976). 2016;41(10):856–63. 10.1097/BRS.0000000000001399 26679887

[46] Jian L, Nagarajan L, de Klerk N, Ravine D, Bower C, Anderson A (2006). Predictors of seizure onset in Rett syndrome. J Pediatr.

[47] Cianfaglione R, Clarke A, Kerr M, Hastings RP, Oliver C, Moss J (2015). A national survey of Rett syndrome: behavioural characteristics. J Neurodev Disord.

[48] Cass H, Reilly S, Owen L, Wisbeach A, Weekes L, Slonims V (2003). Findings from a multidisciplinary clinical case series of females with Rett syndrome. Dev Med Child Neurol.

[49] Reilly S, Cass H (2001). Growth and nutrition in Rett syndrome. Disabil Rehabil.

[50] Halbach NS, Smeets EE, van den Braak N, van Roozendaal KE, Blok RM, Schrander-Stumpel CT (2012). Genotype-phenotype relationships as prognosticators in Rett syndrome should be handled with care in clinical practice. Am J Med Genet A.

[51] Borst H, Weeda J, Downs J, Curfs L, de Bie R (2022). The Rett Syndrome Gross Motor Scale - Dutch Version (RSGMS-NL) can reliably assess gross motor skills in Dutch individuals with Rett syndrome. Dev Neurorehabil.

[52] Schwartzman F, Vitolo MR, Schwartzman JS, Morais MB (2008). Eating practices, nutritional status and constipation in patients with Rett syndrome. Arq Gastroenterol.

[53] Rozensztrauch A, Sebzda A, Smigiel R (2021). Clinical presentation of Rett syndrome in relation to quality of life and family functioning. J Int Med Res.

[54] Lavas J, Slotte A, Jochym-Nygren M, van Doorn J, Engerstrom IW (2006). Communication and eating proficiency in 125 females with Rett syndrome: The Swedish Rett Center Survey. Disabil Rehabil.

[55] Frullanti E, Papa FT, Grillo E, Clarke A, Ben-Zeev B, Pineda M (2019). Analysis of the phenotypes in the Rett networked database. Int J Genom.

[56] Grillo E, Villard L, Clarke A, Ben Zeev B, Pineda M, Bahi-Buisson N (2012). Rett networked database: an integrated clinical and genetic network of rett syndrome databases. Hum Mutat.

[57] Komal Z, Ciara C, Snow B, Hazel F, Daniela T. Rett Syndrome in Ireland: a demographic study. medRxiv. 2023:2023.02.13.23285763.

[58] Neul JL, Benke TA, Marsh ED, Lane JB, Lieberman DN, Skinner SA (2023). Distribution of hand function by age in individuals with Rett syndrome. Ann Child Neurol Soc.

[59] Kadam SD, Sullivan BJ, Goyal A, Blue ME, Smith-Hicks C. Rett syndrome and CDKL5 deficiency disorder: from bench to clinic. Int J Mol Sci. 2019;20(20). 10.3390/ijms20205098 PMC6834180 31618813

[60] Schultz RJ, Glaze DG, Motil KJ, Armstrong DD, del Junco DJ, Hubbard CR (1993). The pattern of growth failure in Rett syndrome. Am J Dis Child.

[61] Buie T, Campbell DB, Fuchs GJ, III, Furuta GT, Levy J, VandeWater J, et al. Evaluation, diagnosis, and treatment of gastrointestinal disorders in individuals with ASDs: a consensus report. Pediatrics. 2010;125(Supplement_1):S1-S18. 10.1542/peds.2009-1878C 20048083

[62] Tarquinio DC, Motil KJ, Hou W, Lee H-S, Glaze DG, Skinner SA (2012). Growth failure and outcome in Rett syndrome. Neurology.

[63] Cianfaglione R, Clarke A, Kerr M, Hastings RP, Oliver C, Felce D (2016). Ageing in Rett syndrome. J Intellect Disabil Res.

[64] Motil KJ, Morrissey M, Caeg E, Barrish JO, Glaze DG (2009). Gastrostomy placement improves height and weight gain in girls with Rett syndrome. J Pediatr Gastroenterol Nutr.

[65] Lai YYL, Wong K, King NM, Downs J, Leonard H (2018). Oral health experiences of individuals with Rett syndrome: a retrospective study. BMC Oral Health.

[66] Roende G, Ravn K, Fuglsang K, Andersen H, Vestergaard A, BrøNdum-Nielsen K (2011). Patients with Rett syndrome sustain low-energy fractures. Pediatr Res.

[67] Sarajlija A, Djuric M, Tepavcevic DK, Grkovic S, Djordjevic M (2013). Vitamin D Deficiency in Serbian Patients With Rett Syndrome. J Clin Endocrinol Metab.

[68] Scully H, Laird E, Healy M, Crowley V, Walsh JB, McCarroll K (2022). Low socioeconomic status predicts vitamin D status in a cross-section of Irish children. J Nutr Sci.

[69] Motil KJ, Barrish JO, Lane J, Geerts SP, Annese F, McNair L (2011). Vitamin D deficiency is prevalent in girls and women with Rett syndrome. J Pediatr Gastroenterol Nutr.

[70] Zhang T, Sidorchuk A, Sevilla-Cermeño L, Vilaplana-Pérez A, Chang Z, Larsson H, et al. Association of Cesarean Delivery With Risk of Neurodevelopmental and Psychiatric Disorders in the Offspring: A Systematic Review and Meta-analysis. JAMA Network Open. 2019;2(8):e1910236-e. 10.1001/jamanetworkopen.2019.10236 PMC6716295 31461150

